# Fever, Intermittent

**Published:** 1854-09

**Authors:** 


*Fever, Intermittent.*—Dr. Harting (Schmidt's Jahrb., 1853, ix.) has employed quinidine with alcohol and sulphuric ether in ague, and, from twelve years experience, states that it is superior to common quinine. He considers the quinidine to be an amorphous quinine, (an opinion which has been strongly opposed by Muller.)—*L' Union Méd.*, from *Southern Med. and Surg. Journ.*

